# Longitudinal measurement of airway inflammation over one year in children and adults with intermittent asthma

**DOI:** 10.1186/1756-0500-7-925

**Published:** 2014-12-17

**Authors:** Frauke Pedersen, Olaf Holz, Frank Kanniess, Stefan Zielen, Johannes Schulze, Adrian Gillissen, Andrea von Berg, Dietrich Berdel, Jutta Beier, Kai Beeh, Maike Schnoor, Helgo Magnussen

**Affiliations:** LungClinic Grosshansdorf, Airway Research Center North (ARCN), German Center for Lung Research, Wöhrendamm 80, 22927 Großhansdorf, Germany; Pulmonary Research Institute at LungClinic Grosshansdorf, Wöhrendamm 80, 22927 Großhansdorf, Germany; Fraunhofer ITEM, Biomedical Research in Endstage and Obstructive Lung Disease Hannover (BREATH), German Center for Lung Research, Feodor-Lynen-Str. 15, 30625 Hannover, Germany; University Hospital Frankfurt, Center for children and adolescents, Goethe University, Theodor-Stern-Kai 7, 60590 Frankfurt am Main, Germany; Robert Koch Klinik, Pneumologie Zentrum, Nikolai-Rumjanzew-Str. 100, 04207 Leipzig, Germany; Marienhospital Wesel, Klinik für Kinder und Jugendmedizin, Pastor-Janssen-Str. 8-38, 46483 Wesel, Germany; insaf - Respiratory Research Institute GmbH, Biebricher Allee 34, 65187 Wiesbaden, Germany; Department of Social Medicine, University Medical Center Schleswig-Holstein, Ratzeburger Allee 160, house 50, 23552 Lübeck, Germany; Practice for Allergy and Family Medicine, Raiffeisenpassage 15, 23858 Reinfeld, Germany; Department of Pulmonary Medicine, Hospital Kassel, Mönchebergstr. 41-43, 34125 Kassel, Germany

**Keywords:** Airway inflammation, Intermittent asthma, Exhaled nitric oxide, Airway hyperresponsivness, Induced sputum

## Abstract

**Background:**

Asthma is an inflammatory disease of the airways, but in clinical practice inflammation is rarely monitored. The aim of this study was to assess the level of airway inflammation in steroid naïve adult and pediatric patients with intermittent asthma over one year.

**Methods:**

54 children and 50 adults with intermittent asthma (GINA step 1) were included. On up to 6 visits lung function, airway hyperresponsiveness to methacholine (PC_20_FEV_1_), sputum eosinophils and exhaled nitric oxide (FeNO) were assessed.

**Results:**

36 pediatric and 34 adult patients were able to produce at least three adequate sputum samples over the study period and were included into the analysis.

In 8 children (22%) the percentage of sputum eosinophils was always below 2.5%. A higher level of eosinophils (>2.5%) was found on at least one visit in 16 (44%) and always >2.5% in 12 children (33%). In the adult group the respective numbers were 14 patients (41%) with always low (<2.5%), 17 (50%) with at least once over 2.5% and three patients (9%) were always above the threshold of 2.5% sputum eosinophils.

**Conclusion:**

These results demonstrate that a substantial number of children and adults with intermittent asthma under ß-agonist treatment only, have variable or persistently high levels of eosinophilic airway inflammation. Long-term studies are needed to observe the progression of asthma severity in such patient populations.

**Electronic supplementary material:**

The online version of this article (doi:10.1186/1756-0500-7-925) contains supplementary material, which is available to authorized users.

## Background

Asthma is a chronic inflammatory disease of the airways. The mildest form of the disease, intermittent asthma is characterized by an almost normal lung function (FEV_1_ > 80% pred.), the use of a β-agonist less than once a week (reliever medication) and nighttime symptoms less than twice per month. Peak-flow measurements are generally normal, except in intermittent periods of symptom worsening [1].

It is known from cross sectional studies that patients with intermittent asthma can have raised numbers of sputum eosinophils [2, 3]. However, no longitudinal studies are available showing the level of airway inflammation in these patients over longer periods of time. This data is available for children with mild to moderate and severe asthma only [4]. It has been shown that reducing inflammation can lead to a lower level of basement membrane thickening [5]. It is also known that a treatment regimen aiming to keep sputum eosinophils below 3% reduces the asthma exacerbations [6–8]. In children with asthma this strategy was not as successful [9].

It was the aim of this study to assess the level of airway inflammation in adult and pediatric patients with intermittent asthma over one year. Over this period the cellular patterns of induced sputum, FeNO as markers of airway inflammation as well as PC_20_FEV_1_ and lung function were measured. The patients were under standard supervision of their pneumologist, who treated them based on lung function and symptoms according to GINA guidelines [1]. The pneumologists were blinded to the results of inflammatory parameters obtained in the study centers. So there was no adjustment of treatment based on inflammatory parameters.

## Methods

### Patients

The study was conducted in five German study sites. Fifty adult patients were recruited in Leipzig, Grosshansdorf and Wiesbaden, and 54 pediatric patients in Wesel and Frankfurt. All patients had received a diagnosis of intermittent asthma within 6 months prior to the study (GINA step 1: according to asthma severity GINA 2002; corresponding to controlled asthma, treatment step 1 GINA 2013 [1]). The inclusion criteria were use of beta-agonist <1/week (max. 4 puffs/occasion), nighttime symptoms ≤ 2/month, no exacerbation 4 weeks prior to a visit. The exclusion criteria were mild persistent asthma (GINA step 2), a smoking history of more than 10 pack years, other severe comorbidities, pregnancy. The study was approved by the Ethics Committee of Medical Association Schleswig-Holstein, Bad Segeberg, Germany. All adult patients and the parents of the pediatric patients gave their written informed consent.

### Medication

All patients received salbutamol at the beginning of the study to be used as reliever medication. Subjects were allowed to use cromoglycic acid, antihistamines or topical corticosteroid medication for the treatment of nasal or eye allergic symptoms during allergy season. Antihistamines, if periodically needed, had to be stopped 48 hours and corticosteroid preparations 2 weeks prior to a visit. Patients were also allowed to use inhaled corticosteroids periodically up to 2 weeks before a visit or cromoglycic acid up to 1 week before a visit to relieve seasonal allergic symptoms. Short acting beta-agonists and anticholinergics were not allowed 6 hours, long acting beta-agonist or anti-cholinergics were not allowed 24 hours or 15 days prior to a visit. Oral antihistamines had to be stopped 48 hours prior to a visit. Oral steroids were not allowed throughout the study.

### Study design

**Figure 1:**
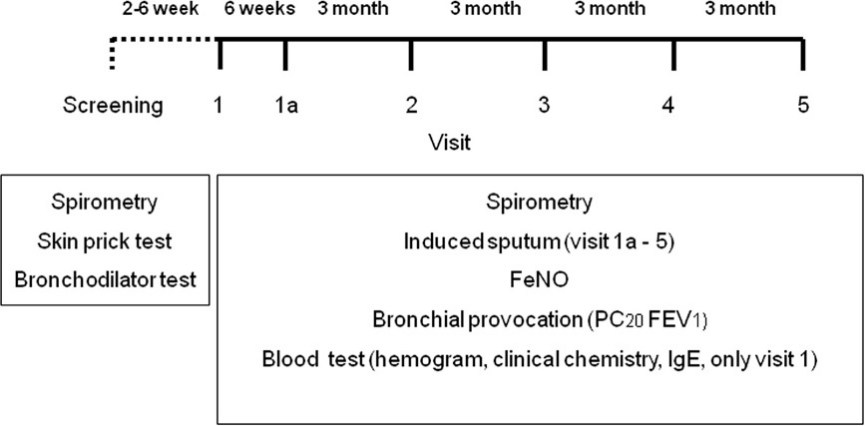
**Study design.**

The study comprised 7 visits over one year (Figure 1). In the pre-study visit (screening), the medical history was assessed and a physical examination, a skin prick test, and a lung function test was performed. A blood sample was taken for clinical chemistry, serum IgE and differential blood cell count on visit 1. On all remaining visits a spirometry, a methacholine challenge, analysis of induced sputum (not visit 1) and a measurement of FeNO were performed.

### FeNO

The level of fractional exhaled NO was detected using a NIOX NO-analyzer (Aerocrine AB, Solna, Sweden). Measurements were performed according to the latest ATS guidelines [10] and all NO-analyzers were calibrated according to the manufacturer’s recommendations.

### Spirometry

Lung function was measured according to the latest ATS guidelines [11] using electronic spirometers (Viasys Healthcare, Höchberg, Germany).

### Sputum induction and analysis

Sputum induction was performed as previously described [12]. Briefly, patients inhaled 3 and 4% nebulized saline solution for two consecutive periods of 5 min each after pretreatment with 200 μg salbutamol. Sputum plugs were selected and pooled from all expectorated material. The sputum processing was performed with Dithiothreitol [13]. Differential cell count were analysed centrally in Grosshansdorf by counting 400 non-squamous cells on Giemsa-stained slides. Results were expressed as percentage of non-squamous cells. The normal value of sputum eosinophils was set to 2.5% according to Spanevello [14].

### Methacholine challenge

Airway hyperresponsiveness was assessed by provocation with nebulized methacholine in increasing concentrations until a 20% fall in FEV_1_ (PC_20_FEV_1_) could be detected [15].

### Statistics

For data with normal distribution mean values and standard deviations, for all other parameters either geometric mean values or medians were calculated. Comparisons between groups were performed by the Wilcoxon Matched Pairs test. The Spearmen correlation coefficient was calculated for the individual comparisons between repeated visits. A p-value below 0.05 was considered as statistically significant.

## Results

### Patients

Patient characteristics are given in Table 1. Out of 104 patients 70 patients (36 pediatric/34 adults) were able to produce three adequate sputum samples over the study period. These patients were included into the data analysis. Additional file 1: Table S1 provides details on the allergy prevalences and Additional file 1: Table S2 on medications and treatments.

**Table 1:** **Patient characteristics**

	Pediatric patients		Adult patients	
n	54	36*	50	34*
gender (m/f)	30/24	21/15	29/21	21/13
age (years)	9.5 ± 2.0	9.9 ± 1.8	34.2 ± 10.4	34,4 ± 9.8
FeNO (ppb)	18.5 (26.1)	19.0 (37.3)	37.1 (69.1)	36.2 (63.3)
FEV_1_ (% pred.)	100.5 ± 12.8	100.7 ± 10.1	99.8 ± 11.7	103.1 (10.4)
PC_20_FEV_1_ (mg/ml)	0.75 (2.4)	1.4 (3.8)	0.9 (2.6)	1.3 (3.8)
IgE (IU/L)	171.5 (342)	152 (332)	188 (320)	199 (285)
Blood Eos (%)	6 (5)	6 (5)	3 (3)	4 (3)

Data of the screening visit separately presented as mean ± SD or median.

(interquartile range; IQR) for all patients and patients (*) able to produce at least on 3.

Visits an adequate sputum sample. m: male, f: female.

### Sputum eosinophils

**Figure 2:**
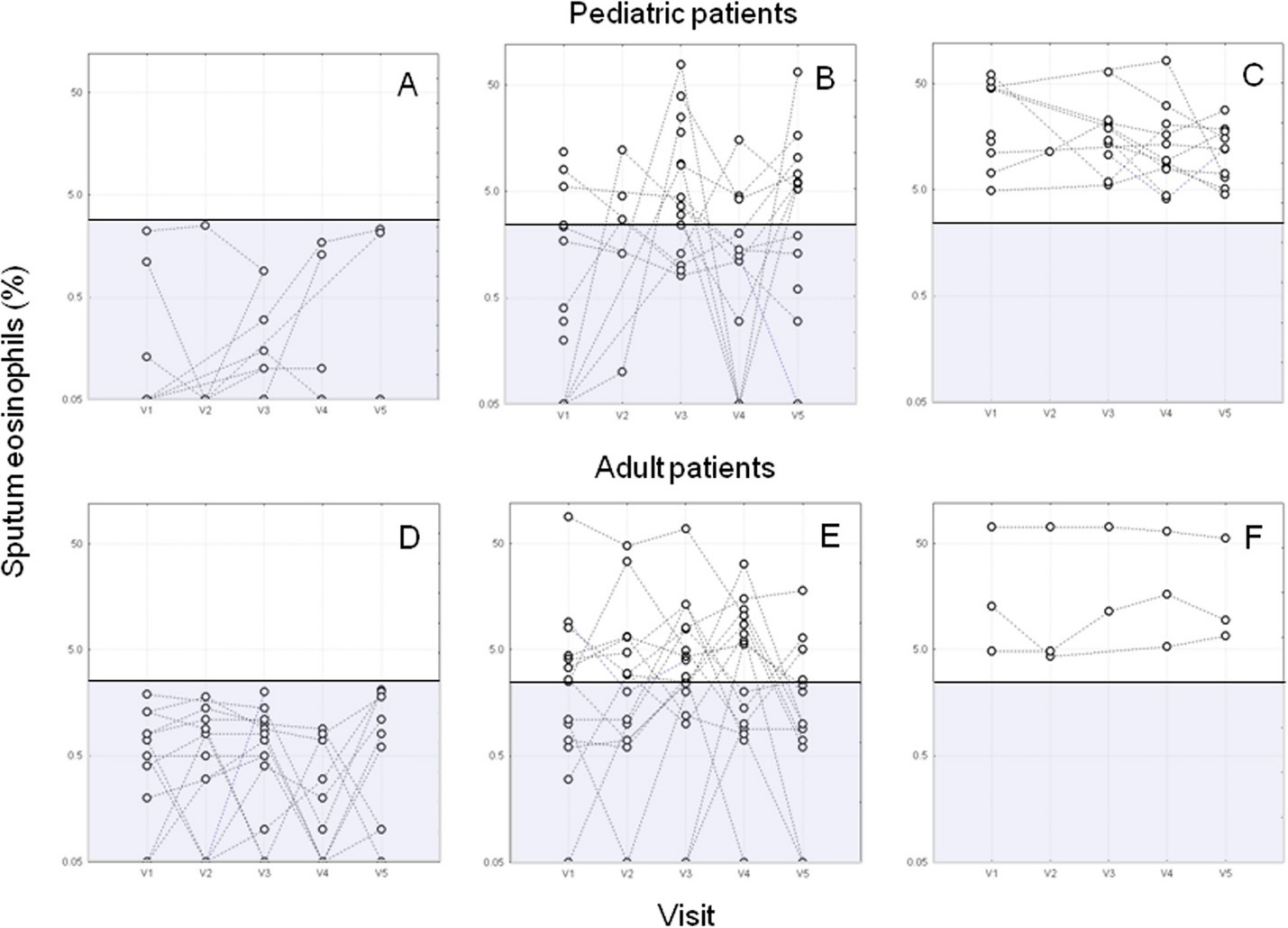
**Percentage of sputum eosinophils over the study period V1 – V5 separately for pediatric and adult patients and for individual patients with always low (A, D), variable (B, E) and persistently high (C, F) percentages of sputum eosinophils.** The cut-off value of 2.5% (shaded area) is displayed. Sputum eosinophils = 0% were set to 0.05 to allow a logarithmic scaling.

We classified both pediatric and adult patients into three groups (“always low”, “variable”, and “always high”) based on the longitudinal sputum eosinophil percentages using a cut-off value of 2.5%. Eight pediatric (22%) and 14 adult patients (41%) showed a low (<2.5%) eosinophil count in all visits. In 16 children (44%) and 17 adults (50%) more than 2.5% eosinophils were found in at least one visit over the study period. For 12 children (33%) and 3 adults (9%) sputum eosinophils were > 2.5% in all visits (Figure 2). The individual values for lung function (FEV_1_) and for the levels of exhaled NO (FeNO) separately for these groups over the study periods are shown in Additional file 2: Figure S1 and Additional file 3: Figure S2.

### Comparisons between patient groups with low, variable and high levels of sputum eosinophils

Additional file 1: Table S3 shows the differences of inflammatory parameters and FEV_1_ between groups and separately for all visits. In adult patients the high eosinophil group also showed significantly higher FeNO values based on the comparison at the different time points. In contrast, the FeNO levels in the pediatric patients did not differ significantly between the three groups.

The FEV_1_ (% predicted) values were not significantly different between groups however, both the children and adults with persistently high eosinophils tended to have the lowest values.

The PC_20_FEV_1_ was significantly lower in adult patients with persistently high eosinophil levels compared to the low eosinophil group, indicating that these adult patients were more sensitive to unspecific airway provocation. Interestingly, the pediatric group with persistently low sputum eosinophils showed the highest level of airway hyperresponsiveness.

Additional file 1: Table S3 also provides the median percentages for sputum neutrophils for the different groups for all visits. Only in very rare occasions the percentage of neutrophils exceeded 50%, so that the neutrophilic asthma phenotype did not play a role in this study.

Additional file 1: Table S4 shows the day and night-time symptoms, the use of rescue medication as well as the peak-flow variability in the morning and in the evening. The data is derived from the diaries of the patients and summarizes the first 7 days following each visit, which were generally not confounded by missing data. Symptoms and use of rescue medication were as low as expected in patients with intermittent asthma, but there was a trend to larger peak-flow variability especially in the adult patients with persistently high eosinophil counts. However, these were only three patients and therefore this result has to be interpreted with care.

Table 2 shows that pediatric as well as adult patients with persistently high eosinophils had significantly higher blood eosinophils at baseline and in pediatric patients there was a trend to higher IgE levels.

**Table 2:** **Comparison between pediatric and adult patients with persistently low, variable and persistently high sputum eosinophils**

		Pediatric patients				Adult patients			
		Low	Variable	High	P	Low	Variable	High	P
Gender	m/f	6/2	12/4	3/9		7/7	11/6	3/0	
Age	years	9,5 (8,0; 11,5)	9 (8,0; 11,0)	10,5 (10,0; 11,0)		31 (27,0; 44,0)	36 (26,0; 38,0)	41 (32,0; 55,0)	
IgE	IU/L	217 (12,0; 898,0)	125 (59,0; 250,0)	175 (88,0; 589,0)		190 (161,0; 411,0)	222 (128,5; 406,5)	75 (64,0; 168,0)	
Allergy#	n	2/4/1/1	4/8/2/2	4/6/2/0		4/3/6/1	5/2/9/1	0/0/2/1	
BloodEosV1	%	4,5 (2,5; 5,0)	6 (4,5; 8,0)	9 (6,5; 10,5)	*	3 (3,0; 4,0)	4 (2,0; 7,0)	8 (7,0; 13,0)**	**

ANOVA: *p < 0.05, **p < 0.01.

#Seasonal/perennial/perennial with seasonal peaks/other or not defined.

### Individual correlations between markers of inflammation

**Figure 3:**
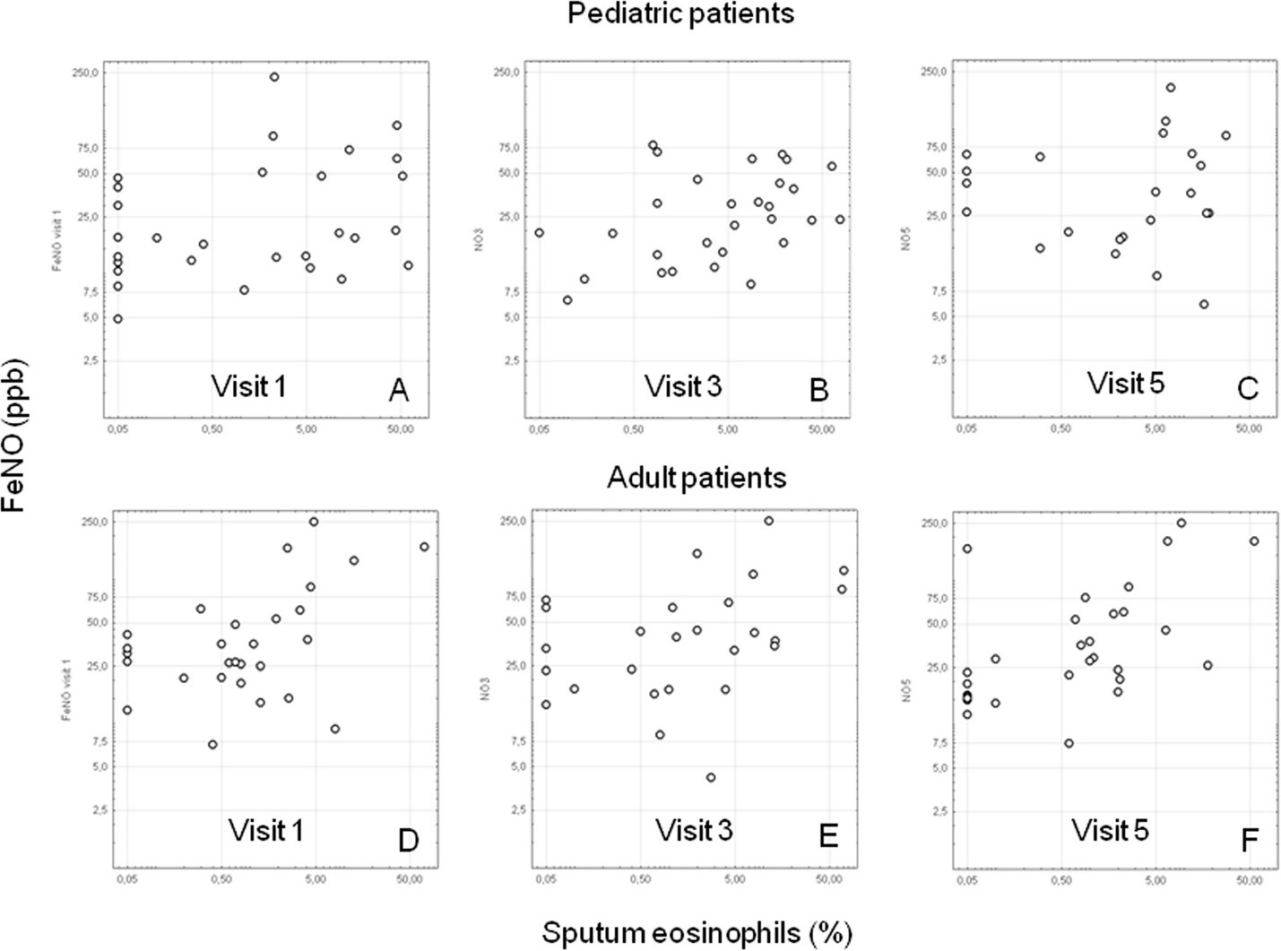
**Correlation between the percentage of sputum eosinophils and the level of exhaled NO (FeNO) for visit 1, 3 and 5, separately for pediatric and adult patients.** Patients with always low **(A, D)**, variable **(B, E)** and persistently high **(C, F)** percentages of sputum eosinophils.

The FeNO levels showed a good to excellent reproducibility over the study period. Furthermore, the correlation coefficients between visits (separate for pediatric and adult patients) ranged from 0.6 to 0.9 and all comparisons were statistically significant (p < 0.001, example in Additional file 4: Figure S3). A similar result was obtained for the comparison of FEV_1_ values between the individual visits. The correlation for PC_20_FEV_1_ was slightly weaker, but significant for all comparisons. As shown in Additional file 4: Figure S3, the reproducibility of eosinophils was weak for about half of all subjects indicating the variability of eosinophilc airway inflammation in these patients.We also looked at the correlation between different inflammatory markers. Figure 3 provides the data for the relationship between sputum eosinophils and FeNO for visit 1, visit 3 and visit 5. There was a slightly better correlation in adult patients.

### Relationship between airway inflammation and symptoms

There were generally only few symptoms in both pediatric and adult subjects (Additional file 1: Table S4). Considering the diary data of the first week following each visit we did not find any significant correlations between symptoms and the different measures of airway inflammation assessed in this study.

## Discussion

In this longitudinal study over one year we found increased levels of sputum eosinophils on at least one occasion in 77% of the pediatric and 59% of the adult patients with intermittent asthma. The three groups with persistently low, variable and persistently high levels of eosinophils differed with respect to FeNO levels and blood eosinophils.

Asthma is defined as a chronic inflammatory airway disease, which is associated with symptoms, airway hyperresponsiveness, and variable limitations in lung function [1]. The phenotypes of asthma are diverse and can be defined by the composition of induced sputum [16, 17]. While neutrophilic asthma is generally more difficult to treat, eosinophilc asthma usually responds well to the treatment with corticosteroids [18, 19]. Therefore, it seems to be rational to measure the extent and the characteristics of airway inflammation in asthma both for diagnostic reasons and to monitor the disease. This is emphasized by studies that adapted the dose of inhaled steroids to keep sputum eosinophils below the normal cutoff-value of 3% and that were able to reduce the exacerbations rate of adult asthmatic patients [6, 7]. While there is one study showing that this strategy is not as successful in children [9], there is also data from the General Practice Research database in the UK showing that increasing the anti-inflammatory treatment as a step-up therapy in uncontrolled asthma is superior in avoiding severe exacerbations and hospital admissions [20]. In a recent review on mild asthma a detailed algorithm for the treatment was presented [21]. However, the diagnostic use of markers of airway inflammation was not mentioned.

Our data demonstrates that in a large number of stable asthmatic patients with nearly normal lung function and with just reliever medication on demand, a significant level of undiscovered eosinophilic airway inflammations exists. For more severe asthma patients who had been under ICS treatment at least periodically a phenotype with persistent eosinophilic airway inflammation has been described before [22]. Raised levels of airway inflammation were also reported by Fleming and coworkers both in adults and pediatric patients with mild to moderate asthma, of whom most (>90%) were under steroid treatment [4]. In line with the largest part of the patients investigated in our study, the inflammatory phenotype was variable over time [4].

We measured lung function, sputum composition, FeNO and airway hyperresponsiveness over a time period of one year. The correlation between visits for FeNO and PC20FEV1 was generally good and statistically significant. For sputum eosinophils this was not true for a large number of patients reflecting the variable sputum levels over time, which has been reported before [4]. Sputum eosinophils and FeNO correlated in the adult patients, which is in line with published data [23, 24]. A larger variability of FeNO values was observed in the pediatric group, most likely due to the fact that FeNO is influenced by body size [10, 25], which is more variable in children and juveniles (in this study from 7 to 14 years) than in adults.

Sputum eosinophils were negatively correlated to the PC_20_FEV_1_ in the adult group, indicating, that in the presence of more airway inflammation, there is generally also a higher degree of unspecific airway hyperresponsiveness [26]. There is evidence that the association between PC_20_FEV_1_ and eosinophilic airway inflammation depends on the duration of the disease [27]. Groenke and colleagues showed that the correlation with eosinophils disappeared with increasing asthma duration and related more to the baseline FEV_1_, possibly reflecting structural alterations in chronic asthma. Unexpectedly, the pediatric group with persistently low eosinophil counts always showed the highest level of hyperresponsiveness. Neither differences in age or in gender distribution (Table 2) can explain this observation. Looking at seasonal and perennial sensitized pediatric patients separately (Table 2) showed a comparable result. Only in perennial sensitized children with additional seasonal symptoms the degree of hyperresponsiveness was similar in the high and low sputum eosinophil groups. However, due to low number these subgroup result need to be interpreted with care.

Our study has some limitations: As often seen in large longitudinal studies, the follow up of patients is sometimes difficult. We also experienced a certain amount of non-adherence to the study protocol resulting in missing values due to missed visits. Another problem was the quality of sputum samples, which was insufficient in a number of cases. Therefore, the assignment to the groups with high and low percentage of eosinophils could not be based on the results of all visits, making also the use of more advanced statistical methods difficult. While all medications were documented (Additional file 1: Table S2), we did not explicitly define and record asthma exacerbations.

It has to be emphasized, that the chosen cutoff-value of 2.5% does not discriminate between healthy and asthmatic patients and that high eosinophil counts can also occur in patients with rhinitis and mirrors airway inflammation during allergen exposure [28]. The comparison between groups with respect to the other markers of inflammation was performed by comparing the results of the individual visits. As the study outline indicates these visits were not performed simultaneously, however, close enough between different groups that such a comparison appeared to be justified.

For pediatric and adult patients with persistent airway inflammation regular bronchodilator treatment throughout most of the study period was sufficient to control symptoms and airflow limitations. Therefore our data raises the question for the need of anti-inflammatory treatment in patients with intermittent asthma. The discussion, whether mild asthma patient should be treated with steroids is old [29, 30]. The results of peak-flow measurements and the record of reliever medication intake collected in the diaries at visit 1 and visit 5 of our study did not provide evidence for a worsening of symptoms in the high eosinophil group. There was also no significant change in FEV_1_ and PC_20_FEV_1_ between visit 1 and 5. However, the already mentioned short term treatment studies [5, 6] would suggest that a risk for more exacerbations and for remodeling within the airways exists. There is also evidence for more airway inflammation in steroid naïve asthma patients that showed a fast decline in lung function over a 5 year period [31]. Furthermore, it has been shown that reducing airway inflammation can lead to an improvement of symptoms [32]. In the Brussel declaration it is emphasized that the knowledge of ongoing inflammation in mild asthma requires the need for short and efficient anti-inflammatory treatment [33].

## Conclusion

The results of our study suggest that including the regular measurement of airway inflammation into the standard monitoring of intermittent asthma patients (GINA step1) can identify patients with variable or even persistent eosinophilic airway inflammation. As some features of severe asthma are attributed to ongoing and potentially to the long history of abnormal inflammatory processes, it appears justified to argue that keeping inflammation as low as possible has the potential for a more beneficial outcome. However, long term prospective studies are required, to resolve this issue.

## Electronic supplementary material

Additional file 1: Table S1: Allergy prevalences - skin prick testing. **Table S2.** Medications: Number of patients with specific treatments. **Table S3.** Comparison of inflammatory parameters and FEV_1_ between pediatric and adult patients with persistently low, variable and persistently high sputum eosinophils. **Table S4.** Comparison of symptoms between pediatric and adult patients with persistently low, variable and persistently high sputum eosinophils. (DOC 118 KB)

Additional file 2: Figure S1: Concentration of FeNO over the study period V1 – V5 separately for pediatric and adult patients and for individual patients with always low (A, D), variable (B, E) and persistently high (C, F) percentages of sputum eosinophils. (TIFF 215 KB)

Additional file 3: Figure S2: FEV1 (% pred.) over the study period V1 – V5 separately for pediatric and adult patients and for individual patients with always low (A, D), variable (B, E) and persistently high (C, F) percentages of sputum eosinophils. (TIFF 192 KB)

Additional file 4: Figure S3: Repeatability of FeNO (A), sputum eosinophils (B) and PC20FEV1 (C) between visit 1a and visit 5. The line of identity is presented in each graph. Circles represent pediatric, squares represent adult patients. (TIFF 100 KB)

## Abbreviations

FeNO: Fractional exhaled nitric oxide
FEV1: Forced expiratory volume in one second
GINA: Global Initiative for Asthma
ICS: Inhaled corticosteroids
IgE: Immunoglobuline E
LABA: Long-acting β_2_-agonist
PC_20_FEV_1_: Provocative concentration causing a 20% fall in forced expiratory volume in 1 s
SABA: Short-acting β_2_-agonist.
