# Supplementary material for: Longitudinal measurement of airway inflammation over one year in children and adults with intermittent asthma
Source: BMC Res Notes. 2014 Dec 17;7:925. doi: 10.1186/1756-0500-7-925 (PMC4301900; doi:10.1186/1756-0500-7-925)
Supplement: Supplementary file 1 — Additional file 1: Table S1: Allergy prevalences - skin prick testing. Table S2. Medications: Number of patients with specific treatments. Table S3. Comparison of inflammatory parameters and FEV1 between pediatric and adult patients with persistently low, variable and persistently high sputum eosinophils. Table S4. Comparison of symptoms between pediatric and adult patients with persistently low, variable and persistently high sputum eosinophils. (DOC 118 KB) [file 13104_2014_3428_MOESM1_ESM.doc]

**Table S3:** Comparison of inflammatory parameters and FEV_1_ between pediatric and adult patients with persistently low, variable and persistently high sputum eosinophils.

Comparison of „low“, „variable“, and „high“ groups by repeated measures (visits) ANOVA separately for pediatric and adults patients. Newman-Keuls posthoc analysis: aa: p<0.01, aaa: p<0.001 for comparison between “high” and “variable”, b: p<0.05, bbb: p<0.001 for comparison between “high” and “low”. Visits with n=1 were excluded from the analysis.

**Table S4:** Comparison of symptoms between pediatric and adult patients with persistently low, variable and persistently high sputum eosinophils

Comparison of „low“, „variable“, and „high“ groups by repeated measures (visits) ANOVA separately for pediatric and adults patients: ns= no significant differences between groups, PFmVK= morning peakflow coefficient of variability, PFeVK= evening peakflow coefficient of variability.

**Table S1:** Allergy prevalence – skin prick testing of all subjects

|  | pediatric patients | adult patients |  |  |
| --- | --- | --- | --- | --- |
|  |  |  |  |  |
| birch | 14/43 (32.6%) | 23/35 (65.7%) |  |  |
| alder | 15/43 (34.9%) | 18/33 (54.5%) |  |  |
| hazel | 15/43 (34.9% | 21/35 (60.0%) |  |  |
| beech | 1/2 (50%) | 14/27 (51.9%) |  |  |
| grass mix | 17/42 (40.5%) | 23/35 (65.7%) |  |  |
| rye | 18/42 (42.9%) | 21/34 (61.8%) |  |  |
| mugwort | 7/39 (17.9%) | 11/32 (34.4%) |  |  |
| ribwort | 5/20 (25%) | 8/32 (25%) |  |  |
| mite (D I) | 18/43 (41.9%) | 13/36 (36.1%) |  |  |
| mite (D II) | 21/42 (48.8%) | 17/36 (47.2%) |  |  |
| cat fur | 8/42 (19.0%) | 17/36 (47.2%) |  |  |
| dog fur | 5/42 (11.9%) | 13/36 (36.1%) |  |  |
| horse fur | 5/42 (11.9%) | 10/36 (27.8%) |  |  |
| Data given as number of subjects tested positive for the respective allergen / total | | | | |
| number of subjects tested (percent tested positive). | | |  |  |

**Table S2:** Medications: Number of patients with specific treatments

|  | pediatric patients | | | | | | adult patients | | | | | |
| --- | --- | --- | --- | --- | --- | --- | --- | --- | --- | --- | --- | --- |
|  |  |  |  |  |  |  |  |  |  |  |  |  |
|  | low | | variable | | high | | low | | variable | | high | |
| total n per group | 8 | | 16 | | 12 | | 14 | | 17 | | 3 | |
| SABA | 6 | | 16 | | 12 | | 10 | | 13 | | 3 | |
| LABA* | 0 | | 0 | | 1 | | 0 | | 1 | | 0 | |
| ICS* | 1 | | 2 | | 3 | | 6 | | 2 | | 1 | |
| ICS/LABA* | 0 | | 1 | | 0 | | 1 | | 4 | | 0 | |
| Mometason (nasal)* | 0 | | 1 | | 0 | | 0 | | 2 | | 0 | |
| anti-histamines* | 2 | | 3 | | 3 | | 4 | | 7 | | 0 | |
| *periodically |  |  |  |  |  |  |  |  |  |  |  |  |
