# Supplementary figures and images for: Longitudinal measurement of airway inflammation over one year in children and adults with intermittent asthma
Source: BMC Res Notes. 2014 Dec 17;7:925. doi: 10.1186/1756-0500-7-925 (PMC4301900; doi:10.1186/1756-0500-7-925)

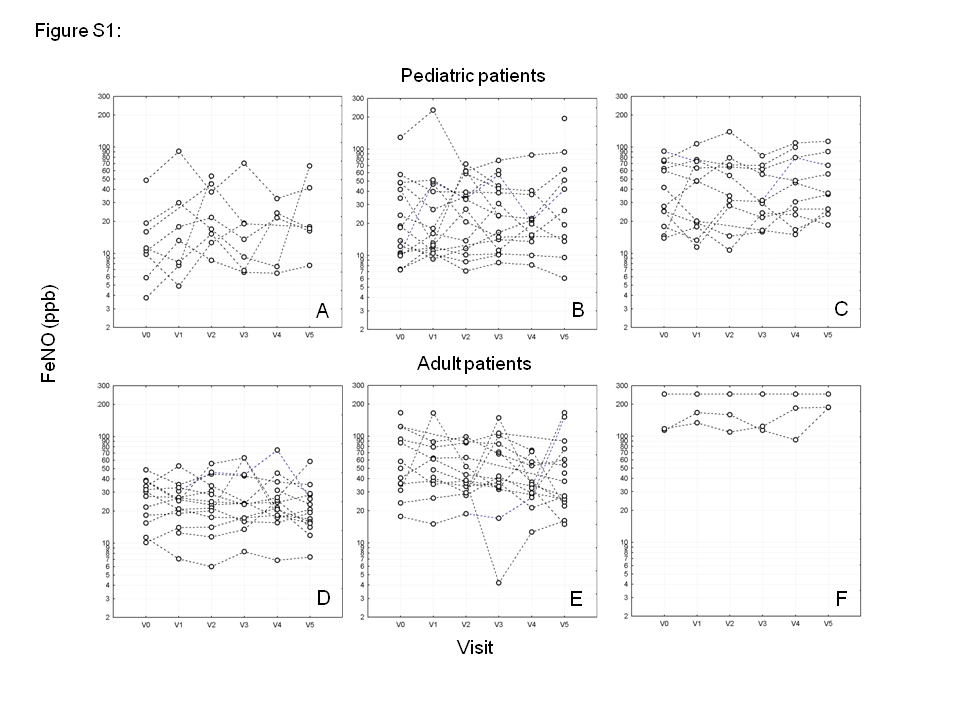

Supplement: Supplementary file 2 — Additional file 2: Figure S1: Concentration of FeNO over the study period V1 – V5 separately for pediatric and adult patients and for individual patients with always low (A, D), variable (B, E) and persistently high (C, F) percentages of sputum eosinophils. (TIFF 215 KB) [file 13104_2014_3428_MOESM2_ESM.tiff]

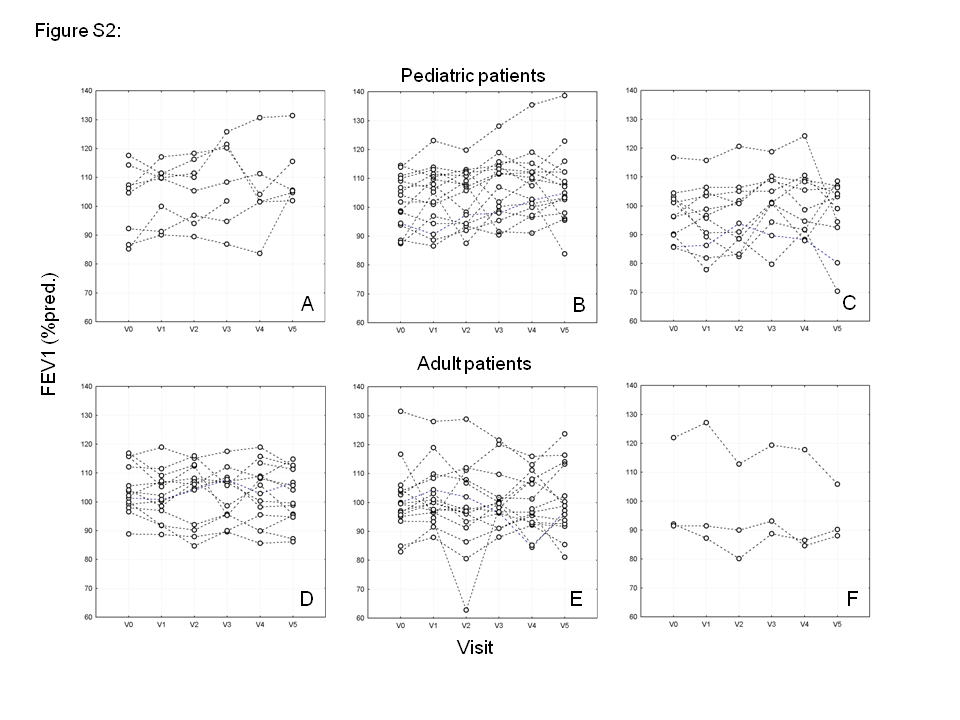

Supplement: Supplementary file 3 — Additional file 3: Figure S2: FEV1 (% pred.) over the study period V1 – V5 separately for pediatric and adult patients and for individual patients with always low (A, D), variable (B, E) and persistently high (C, F) percentages of sputum eosinophils. (TIFF 192 KB) [file 13104_2014_3428_MOESM3_ESM.tiff]

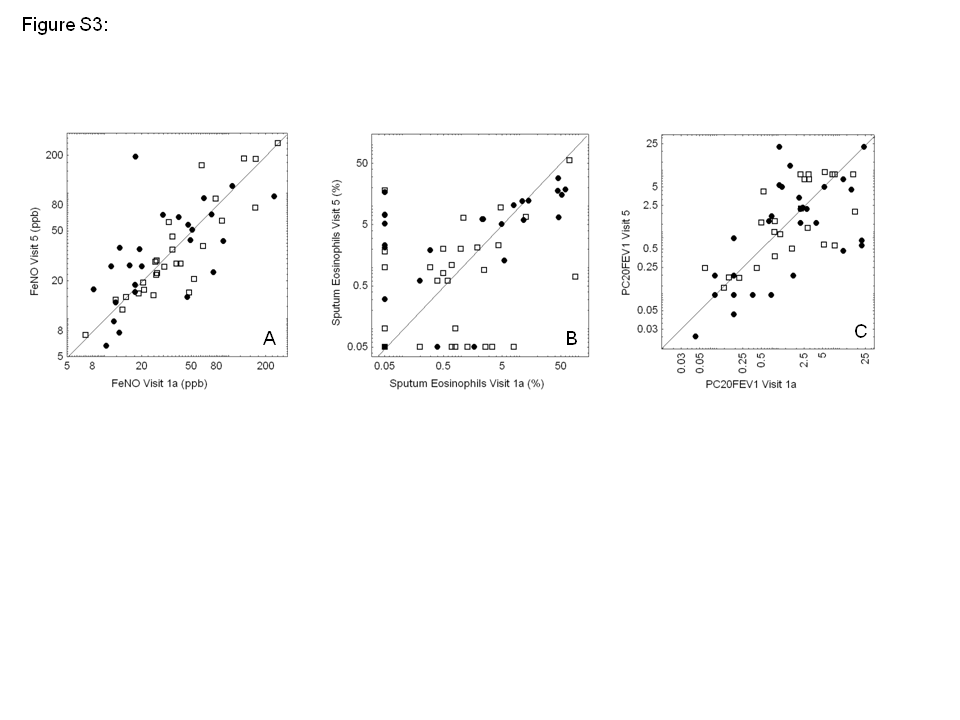

Supplement: Supplementary file 4 — Additional file 4: Figure S3: Repeatability of FeNO (A), sputum eosinophils (B) and PC20FEV1 (C) between visit 1a and visit 5. The line of identity is presented in each graph. Circles represent pediatric, squares represent adult patients. (TIFF 100 KB) [file 13104_2014_3428_MOESM4_ESM.tiff]
